# Development of the Huntington Support App (HD-eHelp study): a human-centered and co-design approach

**DOI:** 10.3389/fneur.2024.1399126

**Published:** 2024-07-01

**Authors:** Pearl J. C. van Lonkhuizen, Anne-Wil Heemskerk, Eline Meijer, Erik van Duijn, Susanne T. de Bot, Jiri Klempir, G. Bernhard Landwehrmeyer, Alzbeta Mühlbäck, Jennifer Hoblyn, Ferdinando Squitieri, Niels H. Chavannes, Niko J. H. Vegt, on behalf of the HEALTHE-RND consortium

**Affiliations:** ^1^Department of Public Health and Primary Care, Leiden University Medical Center, Leiden, Netherlands; ^2^National eHealth Living Lab, Leiden University Medical Center, Leiden, Netherlands; ^3^Huntington Center Topaz Overduin, Katwijk, Netherlands; ^4^Department of Neurology, Leiden University Medical Center, Leiden, Netherlands; ^5^Department of Neurology and Center of Clinical Neuroscience, First Faculty of Medicine, Charles University and General University Hospital in Prague, Prague, Czechia; ^6^Department of Neurology, University Hospital Ulm, Ulm, Germany; ^7^Isar-Amper-Klinikum, Huntington-Zentrum-Süd, Klinik Taufkirchen, Munich, Germany; ^8^St. John of God Hospital, Trinity College Dublin, Dublin, Ireland; ^9^Huntington and Rare Diseases Unit, Fondazione IRCCS Casa Sollievo della Sofferenza Research Hospital, San Giovanni Rotondo, Italy; ^10^Centro Malattie Neurologiche Rare (CMNR), Italian League for Research on Huntington (LIRH) Foundation, Rome, Italy; ^11^Faculty of Industrial Design Engineering, Delft University of Technology, Delft, Netherlands

**Keywords:** Huntington’s disease, neurodegenerative diseases, telemedicine, eHealth, human-centered, quality of life, tele-neurology

## Abstract

**Introduction:**

eHealth seems promising in addressing challenges in the provision of care for Huntington’s disease (HD) across Europe. By harnessing information and communication technologies, eHealth can partially relocate care from specialized centers to the patients’ home, thereby increasing the availability and accessibility of specialty care services beyond regional borders. Previous research on eHealth (development) in HD is however limited, especially when it comes to including eHealth services specifically designed together with HD gene expansion carriers (HDGECs) and their partners to fit their needs and expectations.

**Methods:**

This article describes the qualitative human-centered design process and first evaluations of the Huntington Support App prototype: a web-app aimed to support the quality of life (QoL) of HDGECs and their partners in Europe. Prospective end-users, i.e., HDGECs, their partners, and healthcare providers (HCPs), from different countries were involved throughout the development process. Through interviews, we captured people’s experiences with the disease, quality of life (QoL), and eHealth. We translated their stories into design directions that were further co-designed and subsequently evaluated with the user groups.

**Results:**

The resulting prototype centralizes clear and reliable information on the disease, HD-related news and events, as well as direct contact possibilities with HCPs via an online walk-in hour or by scheduling an appointment. The app’s prototype was positively received and rated as (very) appealing, pleasant, easy to use and helpful by both HDGECs and partners.

**Discussion:**

By involving end-users in every step, we developed a healthcare app that meets relevant needs of individuals affected by HD and therefore may lead to high adoption and retention rates. As a result, the app provides low-threshold access to reliable information and specialized care for HD in Europe. A description of the Huntington Support App as well as implications for further development of the app’s prototype are provided.

## 1 Introduction

Huntington’s disease (HD) is a rare, neurodegenerative disease that is characterized by a gradual progression in motor, cognitive and neuropsychiatric functioning (1–3). HD is caused by an expanded cytosine-adenine-guanine (CAG) repeat in the *huntingtin* gene, which is autosomal dominantly inherited (3). HD gene expansion carriers (HDGECs) often start to experience clinical motor changes between 30 and 50 years of age, leading to a diagnosis of manifest disease (2, 4). Prior to these changes in motor functioning, HDGECs are in the premanifest stage in which they may not experience any symptoms yet (i.e., pre-symptomatic) or can already start experiencing (subtle) changes in motor, cognitive and/or neuropsychiatric functioning (i.e., prodromal) (5, 6). After diagnosis with manifest disease, average lifespan varies between 15 and 20 years (2).

Due to the complex nature of HD and its impact on the quality of life (QoL) of both HDGECs (7) and their partners (8–10), there is a high need for multidisciplinary care services (11, 12) especially in the light of the absence of a cure. Despite the emergence of HD expertise centers across Europe (2, 13, 14), access to these specialty care services is often impeded by the distance of centers (2, 13, 15), health care and transportation costs (2, 13), or physical limitations in later stages of the disease (13, 16). eHealth can overcome these barriers by harnessing information and communication technologies to deliver and/or enhance health care services (17). In this way, eHealth can partially relocate care from specialized centers to the patients’ home (16, 18), thereby reducing travel time and increasing the availability and accessibility of specialty care beyond regional borders.

Although eHealth seems promising for HD, little attention has been paid to eHealth (development) in HD (19–24), especially when it comes to including services specifically designed together with HDGECs and their partners to fit their needs and expectations. Moreover, a recent study suggests that the uptake of telehealth services in HD was relatively low during the COVID-19 pandemic (25). Although the underlying reasons for this limited uptake have not been investigated (25), poor uptake of telehealth services in general is often related to a lack of functionalities desired from users, and/or a poor usability and user experience (26–30). These could be addressed by actively involving end-users in an early stage of the development of such services (18, 26, 31, 32). In other neurodegenerative and neurological diseases (e.g., dementia, Parkinson’s disease, and multiple sclerosis), co-design initiatives have been shown to be beneficial (33–35). Active involvement of end-users is especially important in a disease as complex and rare as HD given the variety in symptoms and needs experienced across disease stages.

We therefore utilized a qualitative human-centered design approach (HCD) (36) by actively involving HDGECs, their partners and health care providers (HCPs) in the development of the Huntington Support App: a web-app aimed to support the QoL of HDGECs and their partners in Europe. This article builds on the previously outlined procedures of the HD-eHelp study (37) by describing the performed development steps and presenting the first versions and evaluations of the prototype of the Huntington Support App. We also describe how we adapted our HCD approach to the challenges we faced while developing the app in the midst of the COVID-19 pandemic and the resulting pressure on the health care systems and HCPs in the participating countries at the time.

## 2 Materials and methods

### 2.1 Study design

The study protocol of this study has been published previously (37). Due to the COVID-19 pandemic, we encountered some delays, which in turn impacted the outlined procedures of the design process in each participating country. As a result, we occasionally had to deviate from the original study protocol. In this methods section, we therefore summarize the key steps of the performed procedures as well as the deviations from the study protocol. For more information on the procedures, we refer to van Lonkhuizen et al. (37).

**Figure 1:**
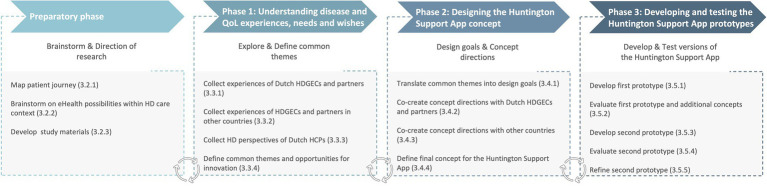
Development phases of the Huntington Support App. QoL, Quality of Life; HD, Huntington’s disease; HDGECs, HD gene expansion carriers; and HCPs, Health care providers. The development process of the Huntington Support App consisted of a preparatory and three design phases. The design was an iterative process of co-designing, evaluating, and adjusting across the different phases. The steps of each phase are described in more detail in the main text. The numbers refer to the numbered subheadings in the main text.

We used a participatory, qualitative human-centered design approach (36) in which end-users participated in all phases of the iterative development process through expert meetings, interviews, focus groups, co-creation sessions, and prototype tests. End-users included HDGECs, partners and HCPs. Figure 1 displays the phases of the development process: (0) preparatory phase; (1) understanding disease and QoL experiences, needs and wishes; (2) designing the Huntington Support App concept; and (3) developing and testing prototypes of the Huntington Support App (37).

This study was part of the HEALTHE-RND project, aimed at enhancing HD care across Europe, conducted between 2019 and 2022. The study was led by the Dutch research team. Research sites from Germany, Czech Republic, Italy, and Ireland also participated. The Dutch research team consisted of professionals with expertise in HD (i.e., senior researcher, psychiatrist, and neurologist) as well as other disciplines (i.e., human-centered design expert, design researcher, neuropsychologist, and health psychologist with expertise in qualitative research and eHealth). The research teams of the other countries included psychiatrists, neurologists, psychologists, and physiotherapists with expertise in HD. Moreover, we established a Dutch advisory board consisting of HCPs from Huntington expertise center Topaz Overduin (i.e., physiotherapist, social worker, elderly care physician, dietician, occupational therapist, speech therapist, team leader, and policy advisor) and an international family patient expert panel (FPEP) [see (37)]. The advisory board, FPEP, and research sites from each country were actively involved throughout the study to enable the app’s suitability within the different healthcare systems and cultural contexts.

### 2.2 Study sample

Premanifest and manifest HDGECs who were 18 years or older and living at home at the time of the study were recruited from the local Enroll-HD databases (38) held at the participating sites (i.e., Leiden University Medical Center, University Hospital Ulm, Charles University/General University Hospital Prague, Fondazione IRCCS Casa Sollievo della Sofferenza Research Hospital Italy, and Bloomfield Hospital/Trinity College Dublin). Partners of premanifest and manifest HDGECs as well as HCPs involved in HD care were recruited via the research site’s primary and secondary care networks (including HD clinics, patient groups, and social media). Sample size was estimated based on guidelines for user testing (39) and differed per stage and country. Dutch end-users were included in all phases of the development process. Due to feasibility and time constraints posed by the COVID-19 pandemic, end-users from the other participating countries were only involved in phase 1, which was less involvement than originally planned (37). To enable the development of an app targeting QoL of HDGECs and their partners across Europe, we considered the collection of end-user experiences (phase 1) in the other countries to be crucial, whereas for the other phases, relying on expert feedback from the other sites was expected to be sufficient.

### 2.3 Study procedures

**Figure 2:**
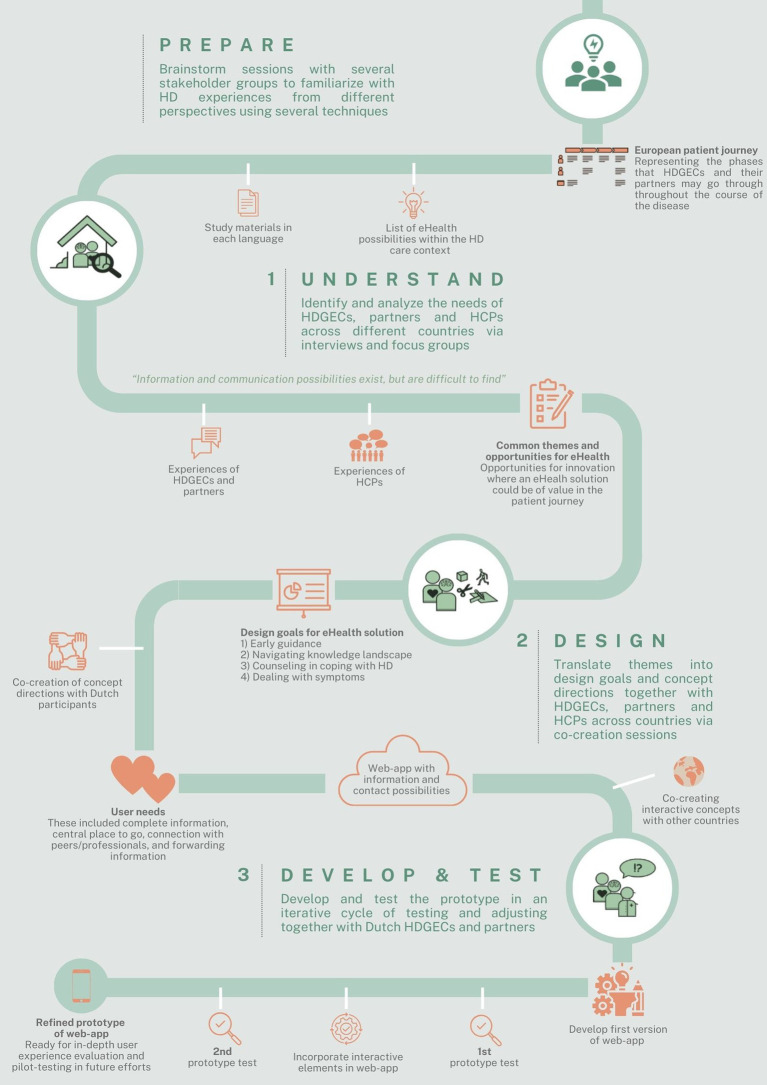
Overview of the iterative development process of the Huntington Support App. HD, Huntington’s disease; HDGECs, HD gene expansion carriers; and HCPs, Health care providers.

Figure 2 provides an overview of the iterative development process. A description of the study procedures per development phase is provided below.

The medical research ethics committee of Leiden Den Haag Delft cleared this study for ethics in the Netherlands (file number: N20.013). For the other participating countries, ethical approval was obtained by the local medical ethics committees at the respective sites. A detailed information letter was provided to all individuals who expressed their interest in this study and informed consent was signed prior to the start of the study activities (37).

#### 2.3.1 Preparatory phase

Meetings were held with the Dutch advisory board to familiarize the researchers with HD experiences from the perspectives of HCPs through patient journey mapping (40). During journey mapping, the advisory board provided input from their expert perspective on different stages that HDGECs may experience during their healthcare journey, including their interactions with human (e.g., family members, HCPs) and non-human actors (e.g., municipalities and information websites). The HD patient journey diagram was checked by the FPEP for cultural and lingual differences and then used to identify promising opportunities for innovation and to gain an understanding of eHealth possibilities within the HD care context. This was done via brainstorm sessions with the advisory board and members of the larger HD community during patient and peer group meetings.

Moreover, the meetings with the advisory board and FPEP set course to the study procedures and materials, including a workbook with sensitizing assignments. These served as preparation to help participants to better express their experiences and needs and were used as reference throughout the study sessions. Both the advisory board and FPEP reviewed the study materials and procedures to ensure comprehensibility and suitability, and to minimize burden on participants. All study materials were translated into the languages relevant for the participating sites.

#### 2.3.2 Phase 1: Understanding disease and QoL experiences, needs, and wishes

Individual semi-structured interviews with Dutch HDGECs and partners were conducted to explore and gather a comprehensive understanding of participants’ daily experiences with HD (caregiving) and their perspectives on eHealth and QoL. The interviews also provided insight in how participants define their own QoL and what HD-related aspects they perceive to affect their QoL (41). This helped in putting participants’ experiences in relation to QoL and eHealth into context in the present article. In addition, we held focus groups with Dutch HCPs about their daily work experience with HD and HD treatment, as well as the challenges and opportunities they face in HD care and their perspectives on eHealth applications (37).

All interviews and focus groups were conducted jointly by a human-centered design expert (NV) and neuropsychologist (PL) via an online videoconferencing platform due to the COVID-19 restrictions at the time, and were audio-recorded and transcribed intelligent verbatim. The other sites collected perspectives of HDGECs, partners and HCPs in their respective countries via digital and/or face-to-face interviews and focus groups.

Transcripts of interviews with Dutch HDGECs and partners were analyzed on themes by two design researchers. To inform the eHealth development process, it was not necessary to analyze all interviews in full detail. Instead, the aim was to gather themes that were mentioned by the majority of interviewees. Interviews were analyzed until no new themes emerged (i.e., thematic saturation) (42). Thematic saturation was defined as the point at which no new themes or insights emerged from the interviews, indicating that additional interviews were unlikely to yield new information relevant to our analysis process. This was the case after coding approximately four interviews in each end-user group (i.e., premanifest HDGECs, manifest HDGECs, partners of premanifest HDGECs, and partners of manifest HDGECs). The remaining interviews were used as contextual background knowledge for the research team to prepare and interpret every participant’s personal contribution in the following phases. The resulting themes were complemented with common themes that emerged from the focus group sessions with HCPs and the themes reported by the other countries. Due to significant delays resulting from the COVID-pandemic, we were unable to perform an in-depth analysis on the transcripts of the other countries as initially planned (37). To include the perspectives of end-users from the other countries, each site analyzed the transcripts on main themes and provided a summary of their findings instead.

#### 2.3.3 Phase 2: Designing the Huntington Support App concept

**Figure 3:**
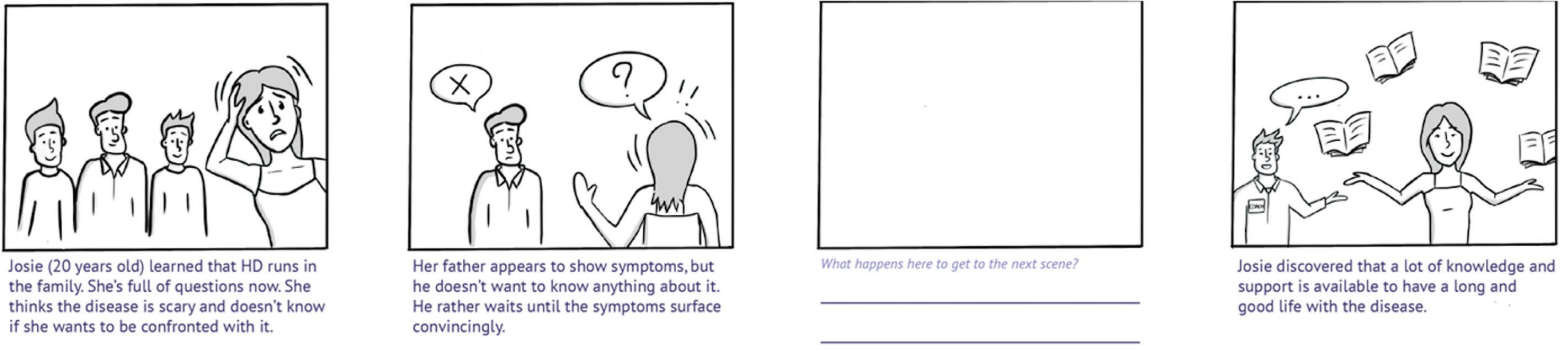
Example of a resulting scenario, included as part of the sensitizing assignments. HD, Huntington’s disease. The scenarios were drawn in simple comic style. Participants were asked to fill in the empty frame based on how they would prefer to go from the problem (frame 2) to a solution (frame 4).

The Dutch research team combined the resulting themes into specific HD-related scenarios that are prototypical for the most common experiences, needs, and wishes of HDGECs and their partners, see Figure 3 for an example. The resulting scenarios were drawn in simple comic style, starting with a frame introducing a particular situation, a frame depicting the problem, an empty frame, and a frame depicting the needed or desired end result. The comics were combined into a printed booklet to serve as the preparatory activity (i.e., sensitizing assignment) for the co-creation sessions. Participants were asked to fill in the empty frame in each comic, which was meant to make them think *how* they would prefer to go from the problem toward the solution.

Co-creation sessions aimed at creating concept directions for the eHealth application were held with the same HDGECs and partners that participated in the previous phase. Followed procedures were inspired by previous work on (co-)design research (43). The co-creation sessions were conducted online by at least two trained researchers and were audio-recorded. In each session, participants discussed several scenarios from the booklet and talked about what would be their ideal experience while a researcher drew new scenarios using a prepared set of PowerPoint props from the scenes-set.^1^

During the development process, we learned that it was not possible to focus on all end-user groups and prioritized HDGECs and their partners as the primary end-users for this study given the remaining time/budget left. HCPs were considered as secondary users and the initially planned co-creation and prototype test sessions with Dutch HCPs (37) were therefore not performed. To ensure the inclusion of HCPs’ perspectives, findings from the co-creation sessions with HDGECs and partners were therefore checked with the advisory board and international team of HD experts. Additional features relevant for international contexts of the eHealth application were subsequently co-created through group sessions with HD experts from the research teams of the other countries.

#### 2.3.4 Phase 3: Developing and testing prototypes of the Huntington Support App

The input from the above-described co-creation sessions was summarized and the final concept direction and system requirements for the prototype of the eHealth application were defined by the most salient needs and wishes that were expressed and prioritized during the sessions with all stakeholders. The final concept direction was translated into specific functionalities through user stories (i.e., short statements of what the eHealth application should do for the user). An app-developer built a web-based app prototype.

Dutch end-users (i.e., HDGECs and partners from this point forward) that participated in the previous phases were invited to test and provide feedback on the first and second working prototype of the app. The prototype was a partially implemented web-app with sufficient content for testing. Participants were instructed to explore the prototype once, either on their phone or computer. In case of the latter, the mobile version was shown on their computer screen. Participants were able to test the prototype from within their home by granting them access to the prototype URL. During the first round (from December 2021 to January 2022), participants filled-in questions on functions and lay-out of the prototype. The questions covered first impressions, uniqueness, pleasantness, and ease of use of the app. Participants rated these questions on a five-point Likert scale (e.g., ranging from 1 = strongly agree to 5 = strongly disagree). Examples of open-ended questions included: “What do you like/dislike most about the app/its functions?” and “Do you have any additional suggestions for improving the app?” Participants were able to share any additional feedback with the research team in subsequent online feedback sessions during which participants were encouraged to share their thoughts on the prototype.

As the process was iterative, the development of the first prototype based on the Dutch input partly ran in parallel with co-creating additional functionalities with experts from the research sites in the other countries. As a result, we were able to combine concept testing, which was originally planned for phase 2 (37), with testing the first version of the prototype. Hence, next to testing the first prototype, Dutch participants were asked to provide feedback on digital mock-ups of potential additional interactive features (such as a walk-in hour or live chat) of the app that resulted from the co-creation sessions with the other countries. In this way, the preferences of the Dutch end-users and those of the experts from the other countries, including the fit with care systems and privacy and security legislation, were combined in deciding on which interactive features were going to be developed in the second version of the prototype. Based on participants’ feedback during the first test, refinements were made to the first version of the prototype. During the second round of prototype testing (from April to May 2022), the same procedures were followed as for the first version, yet with a stronger focus on the newly implemented interactive features.

The subsequent refinements to the second prototype into a final prototype marked the end of the human-centered design process as described in this paper.

## 3 Results

### 3.1 Characteristics of end-users

In the Netherlands, 12 HDGECs (six premanifest, six manifest), 12 partners of HDGECs (six of premanifest and six of manifest HDGECs), and 12 HCPS were included in this study. The HCPs represented a variety of professionals working in Dutch HD expertise centers (see Table 1).

**Table 1:** Sociodemographic characteristics and participation rates of Dutch end-users.

	HDGECs (*n* = 12)	Partners (*n* = 12)	HCPs (*n* = 12)
Age (mean; range)	53; 31–68	46; 25–71	46; 28–68
Gender [*n* (%)]			
Male	6 (50)	8 (67)	8 (67)
Female	6 (50)	4 (33)	4 (33)
HD stage affected individual [*n* (%)]			
Premanifest	6 (50)	6 (50)	-
Manifest	6 (50)	6 (50)	-
HD disease stage (UHDRS-TFC score) (mean; range)			
Premanifest HDGECs	13; 11–13	-	-
Manifest HDGECs	11; 9–13	-	-
Living situation [*n* (%)]			
Together with partner	9 (75)	12 (100)	-
Alone	3 (25)	-	-
Time since genetic test in years (mean; range)	8; 1–18	-	-
Profession [*n* (%)]			
Neurologist	-	-	1 (8)
Social worker	-	-	2 (17)
Dietician	-	-	1 (8)
Physiotherapist	-	-	1 (8)
Psychologist	-	-	2 (17)
Speech therapist	-	-	1 (8)
Occupational therapist	-	-	1 (8)
Elderly care physician	-	-	1 (8)
Elderly care physician and psychiatrist	-	-	1 (8)
General practitioner	-	-	1 (8)
Participation in co-creation session [*n* (%)]	9 (75)	10 (83)	-
Participation in prototype session [*n* (%)]			
Prototype 1	9 (75)	7 (58)	-
Prototype 2	7 (58)	8 (67)	-

HDGECs, Huntington’s disease gene expansion carriers; HCPs, Health care providers; *n*, Number of participants; HD, Huntington’s disease. UHDRS-TFC score: Unified Huntington’s Disease Rating Scale – Total Functioning Capacity score, ranging from 0 to 13 with lower scores indicating more decline in functioning (44). Numbers and percentages are rounded to the nearest whole number.

For the additionally collected experiences of end-users in the other participating countries, the following numbers were recruited: Germany: six HDGECs and five partners; Italy: six HDGECs and six partners; Ireland: seven HDGECs, seven partners, and six HCPs; Czech Republic: no end-users were included (see Supplementary Table 1 for an overview of the end-users characteristics from each participating country).

### 3.2 Preparatory phase

#### 3.2.1 Map patient journey

**Figure 4:**
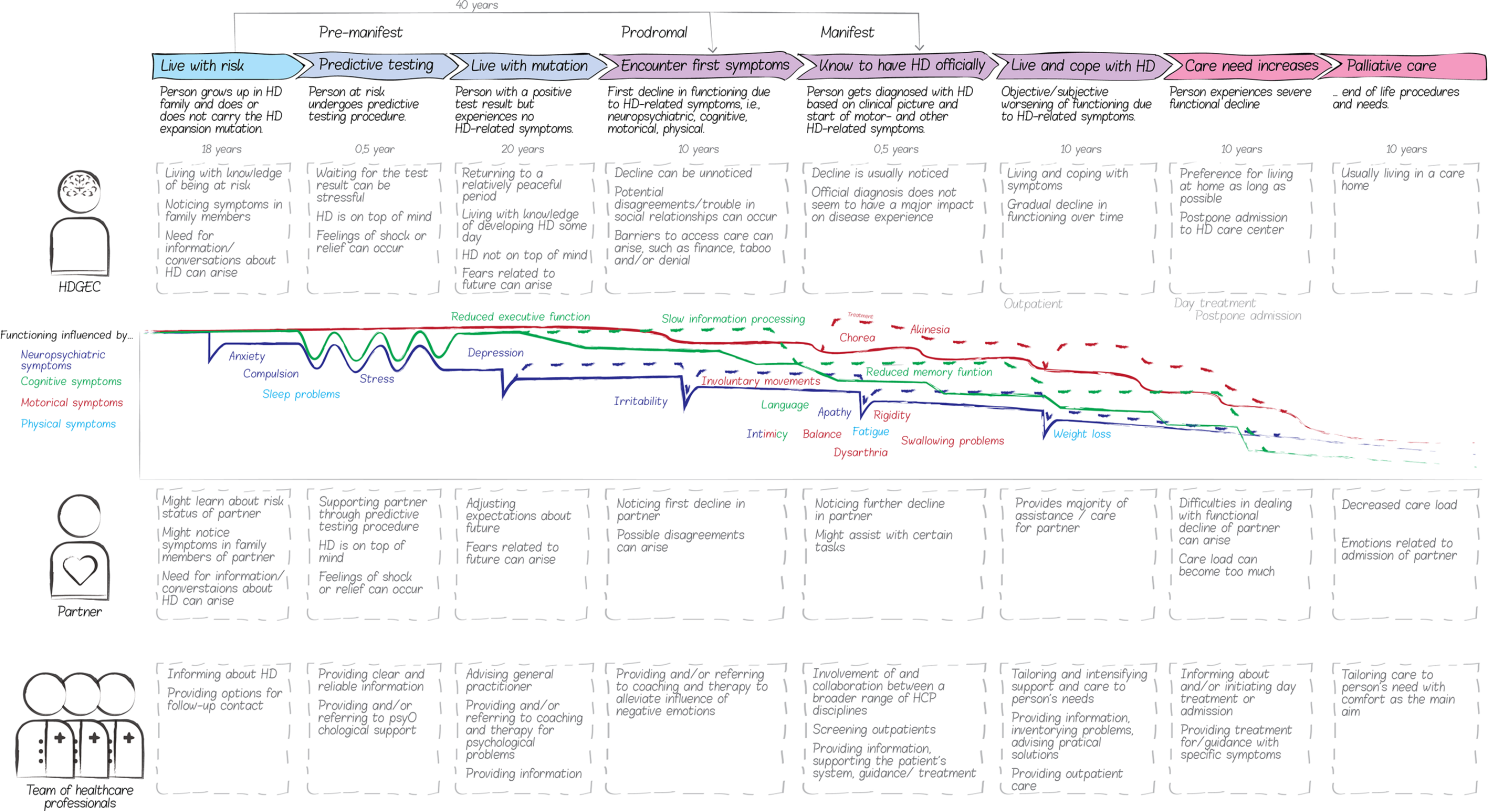
The HD patient journey based on advisory board and panel participants. HD, Huntington’s disease; HDGECs, HD gene expansion carriers; and HCPs, Health care providers. The European patient journey represents the phases that HDGECs and their partners may go through throughout the course of the disease, based on the experience of board and panel participants. The patient journey also depicts the corresponding actors, feelings, and activities relevant for eHealth development.

The creative group sessions with the Dutch advisory board and the FPEP resulted in a visual representation of a European patient journey, consisting of eight phases that, in the experience of board and panel participants, HDGECs and their partners may go through throughout the course of the disease, see Figure 4. It should be noted that this patient journey is a comprehensive visualization of all *possible* phases HDGECs and their partners may go through. It does not represent the common disease experience as HDGECs and/or their partners may not experience all phases in most cases, for instance because the disease is only recognized in later phases or because people at risk may decide not to test.

#### 3.2.2 Brainstorm on eHealth possibilities within HD care context

Brainstorm sessions with several stakeholders led to a comprehensive list of eHealth possibilities that could support QoL, including information provision, communication, and (self)monitoring. Important key features of eHealth according to the advisory board included tailored, remote care that is always and quickly accessible. At the same time, the board emphasized that the quality of information and communication as well as privacy and security should be ensured. Participants at the patient and peer group meetings expressed to have difficulty with finding relevant and reliable information on the disease and HD-care services due to its diffusion across the internet. Moreover, they indicated that several digital tools are already available, yet not tailored to HD.

#### 3.2.3 Develop study materials

Several study materials were developed, including sociodemographic questionnaires, interview/focus group protocols, and workbooks with sensitizing assignments (see (37) for the workbook of phase 1). Separate workbooks were developed for premanifest and manifest HDGECs each, and subsequently pilot tested. This resulted in some important changes to the workbook for manifest HDGECs that have been described previously (37).

### 3.3 Phase 1: Understanding disease and QoL experiences, needs, and wishes

#### 3.3.1 Collect experiences of Dutch HDGECs and partners

Through the sensitizing assignments and interview data, we collected disease experiences, and needs and wishes from Dutch HDGECs and partners. During the analysis, the research team gathered a broad understanding of participants’ personal experiences with the disease, their symptoms, coping strategies, and specific moments in their lives when HD had a major impact. A major topic that came forward in most interviews was how the disease influences social relationships. Most participants talked about the challenges they faced concerning talking about the disease with others, and how the symptoms may or already were influencing their contact with friends, family, and colleagues.

The needs and wishes that were expressed mostly involved references to staying active and being able to live at home. Another recurring wish expressed the commonly shared hope for a medicine that could cure HD. Most HDGECs and partners did not mention any digital tools and mentioned that not much is available specifically for HD. Other participants were quite well informed and explained that there are some useful digital tools, such as agendas and timers, yet the online information about HD is for most interviewees difficult to find as they found it to be very scattered.

#### 3.3.2 Collect experiences of HDGECs and partners in other countries

For the other participating countries most of the findings overlapped, yet we also found variations. In Germany, HDGECs and partners mentioned financial stability more often compared to Dutch participants. They were more concerned with staying at work and having insurance to cover health costs. They also expressed the wish for more HD-expertise among healthcare professionals, and taboo and stigmatization were strong barriers for them to talk about HD with anyone, even family. Participants in Ireland and Italy often mentioned taboo, stigma and guilt, as well as lack of support and resources in the care system. Italian participants often expressed the need for contact with peers.

#### 3.3.3 Collect HD perspectives of Dutch HCPs

During the three focus group sessions, with four Dutch HCPs each, most indicated that they were involved in later phases of the journey and only social workers were involved throughout the complete journey.

The discussed topics included reduced disease awareness by manifest HDGECs, care avoidance, and the current HD care path in the Netherlands. This helped the research team to better understand HD care and challenges that HCPs struggle with. Moreover, it provided the research team a broader perspective to better position the stories of HDGECs and their partners and would later help in deciding on functionalities and defining an implementation strategy of the to-be-developed eHealth application.

Regarding the use of eHealth, HCPs mainly mentioned which digital information and communication tools already exist. In contrast with HDGECs and their partners, HCPs explained that a lot is already available and adding something new might be a challenge. They, for example, mentioned online training videos, information resources, and tele-care technologies. A common remark at the end of a focus group was that they “liked to hear about experiences from colleague HCPs with expertise in HD,” suggesting that strengthening the coordination and communication between HCPs would be an interesting direction for applying eHealth as well.

#### 3.3.4 Define common themes and opportunities for innovation

From the conversations with Dutch end-users, we concluded that many digital information resources, communication possibilities, and supportive tools exist yet most participants have a hard time finding them. Especially in the earlier phases of the disease, when people do not have much contact with HCPs yet, HDGECs and their partners often suffer from lack of information.

**Figure 5:**
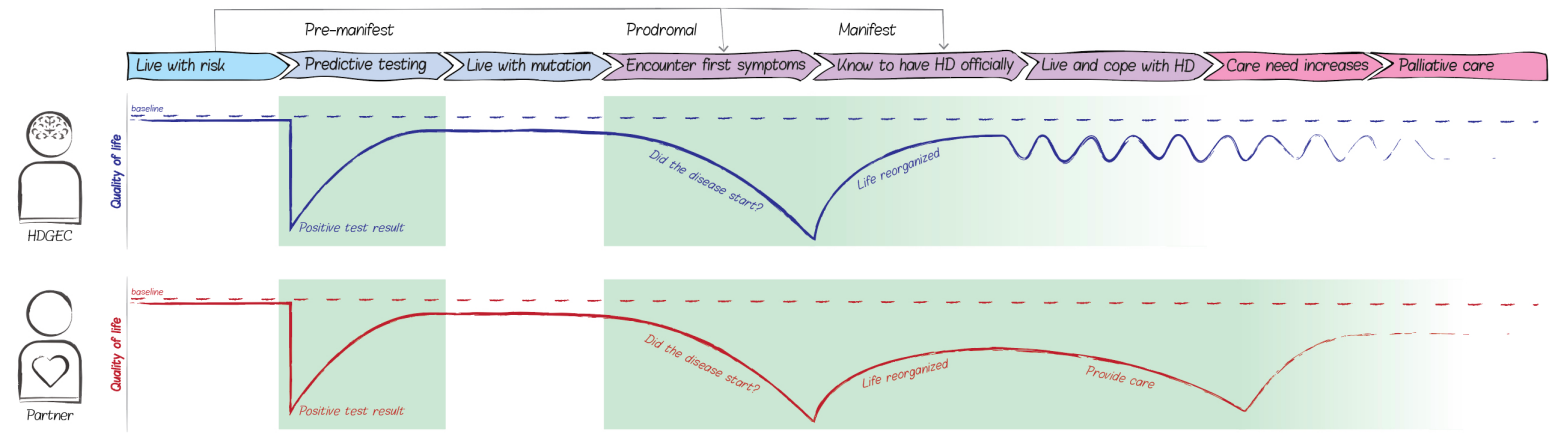
Opportunities for innovation within the patient journey map. HD, Huntington’s disease. The decreases in quality of life mark the identified opportunities in which an eHealth solution could contribute to the quality of life of HDGECs and their partners. Two phases in the patient journey for HDGECs and three phases for partners were identified where QoL is impaired, and an eHealth solution could be of value.

Two phases in the patient journey for HDGECs and three phases for partners were identified as opportunities for innovation, i.e., where QoL can be significantly impaired and an eHealth solution could be of value (see Figure 5). Receiving a positive predictive test result can lower people’s QoL due to a major shift in one’s expectations for the future. In this phase, reliable, clear and person-centered information early on might alleviate a decrease in QoL. We also identified a possible decrease in QoL in later phases, when the first symptoms set in and people are often very attentive to symptoms and wonder whether the disease has truly started. HDGECs and partners may start to look for more practical knowledge regarding dealing with specific situations and symptoms to keep on living an autonomous, active and social life and keep on living at home as long as possible. In other cases, when people have not had a predictive test, the first symptoms are often very impactful on one’s daily functioning. Maladaptive coping strategies, such as avoiding conversations about the disease, and the taboo and stigma surrounding HD may complicate care provision. The anonymous nature of eHealth could help with such complications. Therefore, providing support at the time of discovering first symptoms could significantly improve HDGEC’s and partner’s QoL. Among partners, we identified a possible third decrease in QoL when the informal caregiver role requires too much from them. In this phase, partners may benefit from (digital) contact with peers and caregivers.

### 3.4 Phase 2: Designing the Huntington Support App concept

#### 3.4.1 Translate common themes into design goals

The knowledge gathered in the preparatory and understanding phase with Dutch participants was translated into four design goals by the research team: (1) early guidance, (2) navigating through the knowledge landscape, (3) counseling in coping with HD, and (4) dealing with symptoms. These goals were based on the decision to primarily focus on HDGECs and their partners as end-users, and formulated based on the major and common themes that they mentioned.

“Early guidance” relates to the finding that the disease is often still a taboo subject and barriers have to be overcome in order to talk about it and find help and support. Digital resources can play an important role in providing carefully structured information, allowing users to manage what information they want to see and what not. Providing a tool to “navigate through the knowledge landscape” is based on the finding that online HD-related information is very scattered and the need for tailored, nuanced information that addresses users’ specific needs. Digital tools can facilitate this by offering clear navigation structures that distinguish between theoretical and practical information, ensuring relevance for different target groups. The goal of providing “counseling in coping with HD” through digital tools relates to the different coping strategies that participants discussed in relation to dealing with HD. Digital tools can support these strategies by offering low-threshold contact with HCPs. The goal of “dealing with symptoms” through digital tools came from the realization that there are already all kinds of resources that help deal with the symptoms of HD. For this, however, centralizing commonly used tools and providing referrals for specific complaints can aid individuals in effectively managing their symptoms. For more information about the design goals, the Supplementary material can be referenced.

#### 3.4.2 Co-create concept directions with Dutch HDGECs and partners

In total, four co-creation sessions were held, one with each Dutch end-user group (i.e., premanifest HDGECs, manifest HDGECs, partners of premanifest HDGECs, and partners of manifest HDGECs). Five participants (two partners and three HDGECs) were unable to attend the session due to summer holidays (*n* = 2), other obligations (*n* = 2), or not wanting to partake in a group video call (*n* = 1, partner of manifest HDGEC).

In these sessions, the four above-described design goals were explored by creating small stories within predefined scenarios that represented the goals. In general, the stories from the participants were about two basic functionalities that an eHealth solution for HD should have: information and contact possibilities. The different groups had varying wishes regarding these functions, such as more generic information about HD for the pre-manifest groups and specific information about care and coping for the manifest groups. Regarding contact possibilities, all groups wished for informal and formal contact options to exchange experiences and find help. The manifest HDGEC group mentioned supportive tools as an additional functionality in the eHealth application, such as therapy instructions and an agenda with a program of activities.

Discussions during the co-creation sessions led to four overall user needs that HDGECs and partners have concerning eHealth solutions reflecting the concept directions of information and contact possibilities. First, they want complete and correct information about HD. Secondly, they wish for one place where all available professional and informal support is listed. The third user need was to enable an actual connection with a professional or peer. The final user need was information for friends and family that HDGECs or partners themselves can forward, which could help them in “fighting” to be understood and letting others know how they are doing.

#### 3.4.3 Co-create concept directions with other countries

HD-experts and patient representatives from the other participating countries indicated that HD expertise is either scarce or scattered across the country. The experts from the Czech Republic mainly expressed interest in discussion groups among professionals to improve HD-expertise in the care system and a “help-line” for HDGECs and partners. A good feature of a new information platform would be that HCPs themselves can easily update the information, because the knowledge about HD is developing rapidly. In Germany, there was the wish to better centralize HD knowledge and spread HD expertise across the country. Yet this was considered difficult because there are several autonomously operating care regions. Hence, a central location for information and contact, such as streaming events and online group sessions, was desired yet challenging to organize. For the Irish team, the eHealth solution was mainly seen as a medium to put HD more on the map as expertise in HD is scattered across the country. A central digital platform would thus be a helpful medium in Ireland to increase awareness among policy makers and HCPs. Additionally, the international collaboration on the platform would help Irish end-users because the Irish care system is not well organized for HD and could benefit from the knowledge and expertise of other countries. In Italy, the main interest was in a telemedicine functionality, to overcome the large distances in the country. This would mainly help the HD expertise center in distributing their expertise across the country. Hence, functionalities such as video consultation, home monitoring, and consultation booking, were expected to be interesting features for Italian end-users according to the experts from the Italian research site.

Based on the gathered information, some potential additional interactive features were envisioned, including an online walk-in hour in which participants could ask questions to an HCP, the possibility to book an appointment with an HCP for an online or in-person visit, a live chat function to ask questions directly, and a contact form.

#### 3.4.4 Define final concept for the Huntington Support App

Based on the findings from all participatory activities as described above, the research team defined a final concept for the eHealth solution, i.e., a web-app that should serve as a trusted safety net to support the QoL of people affected by HD. This app should function as an HD helpdesk with understandable general information, answers to frequently asked questions, and referral/contact information. The general information should provide a simple, compact, and reassuring description of HD for outsiders and a detailed description with a patient journey, conditions for care, and referrals for people directly affected by the disease. Specific information should be provided through a Q&A-like database, including descriptions of symptoms, care pathways, and information tailored to specific situations. Contact information should support online and offline connections with peers, family and friends, and HCPs. And ideally, the app should contain tools, such as a calendar with events and scientific studies that people can participate in, a personal agenda with day and week schedule, therapy instructions, and a quick contact button for crisis moments. Considering these components, the app could be of value in the previously identified phases in the patient journey map (as described under the Section 3.3.4).

### 3.5 Phase 3: Developing and testing prototypes of the Huntington Support App

#### 3.5.1 Develop first prototype

To increase accessibility, it was decided to build a web-app that is accessible on different devices (e.g., phone, computer, and tablet). This was considered important as it circumvents potential difficulties with downloading or logging into the app once motor and/or cognitive decline sets in, and, at the same time, allows for the use of custom devices if they have a web browser.

**Figure 6:**
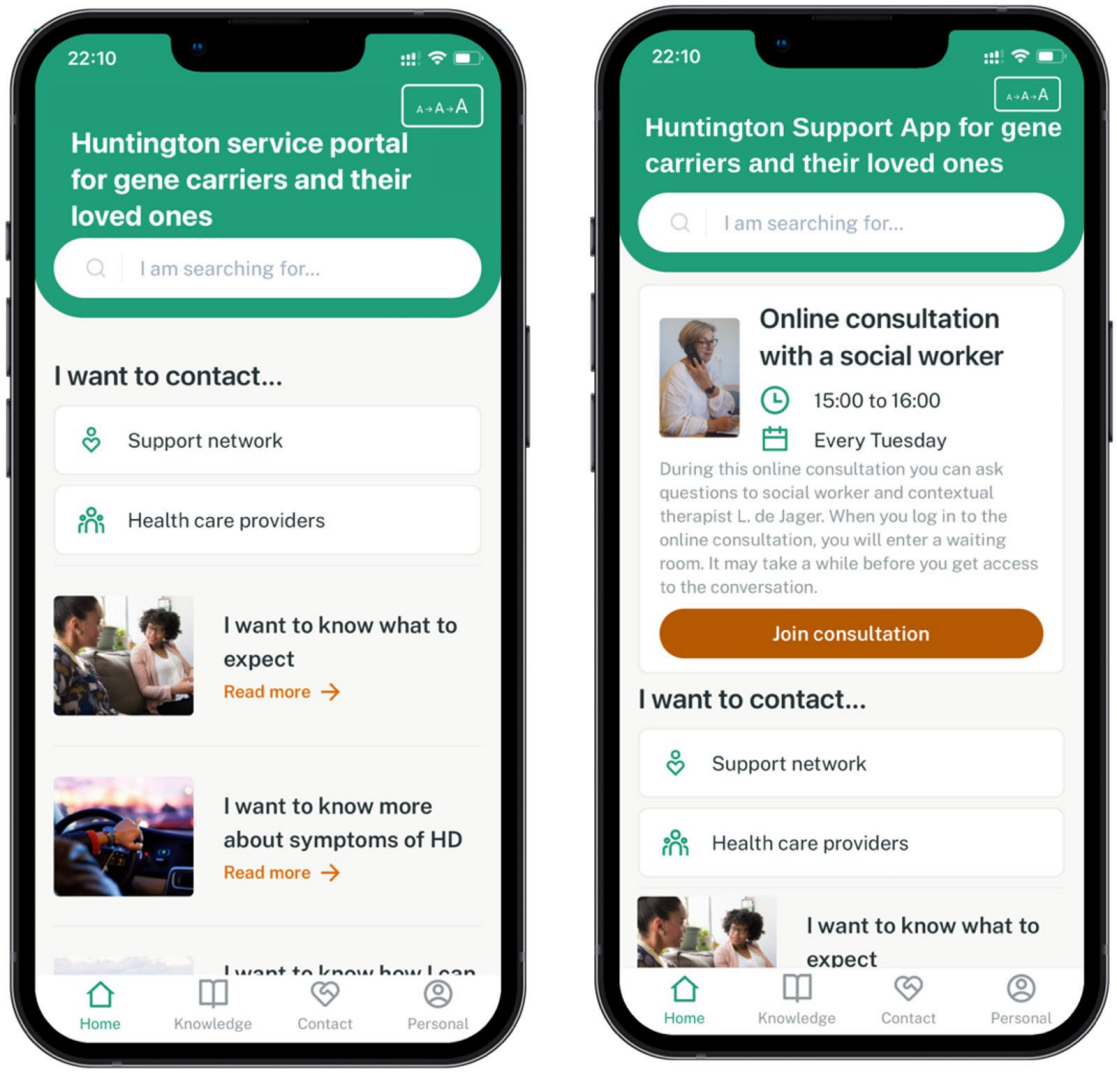
Home page of the first (left) and final (right) prototype of the Huntington Support App. The print screens of both prototypes were translated to English for this article. Prototype testing was performed with the Dutch versions of the prototypes. The first version of the home page of the prototype (left) included two functionalities: information and contact possibilities. In the final version of the prototype (right), interactive features to the app were added, including the online walk-in hour on the home page.

The first version of the so-called “Huntington Service Portal” at the time, is displayed in Figure 6. The first version of the web-app was based on two main concept ideas from the previous phase, i.e., information and contact possibilities. The following components were implemented in the first version: (1) Home page, consisting of featured articles, quick access to contact information from peers and HCPs and the option to change text size; (2) Knowledge repository, including structured information on HD in general, symptoms, specific situations that individuals might encounter (e.g., housing and financial situation, concerns about having children at risk), and news articles about HD; (3) Contact page, consisting of an overview of all national HD centers and experts categorized per discipline, as well as an overview of future HD events (e.g., peer groups, Huntington café’s) and experience stories of peers; and (4) Personal page, including a privacy statement, the possibility to get in contact about the app and to change preferences regarding text size and region (i.e., the Netherlands, England, Czech Republic, Germany, and Italy). As the app provides a clear and layered navigation structure, users can easily decide on which information they would like to see.

In parallel, a content management system was developed to manage the content of the web-app in each country. The initial structure of the content (i.e., topics and headings) and some examples were drafted together with the Dutch advisory board and was considered sufficient for prototype testing. Content was written from the point of view of either the HDGEC or partner as end-user (e.g., “I experience” instead of “the patient may experience”) in a clear and simple way and tailored to specific situations HDGECs (e.g., information on mortgage, child wish) and/or partners (e.g., care role, intimacy) may encounter.

With regard to the envisioned interactive features in the co-creation sessions with the other countries, digital mock-ups were drafted to gather participants’ feedback on the concepts of these potential features during prototype testing.

#### 3.5.2 Evaluate first prototype and additional concepts

In total, 16 of the Dutch participants that participated in the previous phases tested the first version of the prototype and provided their feedback (i.e., five premanifest HDGECs, four manifest HDGECs, four partners of premanifest HDGECs, and three partners of manifest HDGECs).

The initial reaction of all participants to the first version of the prototype was (very) positive (*n* = 16). More particularly, most participants rated the app as (very) appealing (*n* = 14), pleasant (*n* = 14), and easy to use (*n* = 15). Participants indicated that the app bundled all relevant information on HD. A partner of a manifest HDGEC wrote: *“[The app is] built from/based on the demand of the ‘customer’. And especially: everything is now finally clearly bundled together.”* Participants additionally commented that the app consisted of correct, clear and the right amount of information about the disease, and valued how the information of the app was arranged and also tailored to a specific situation someone is in. A slight majority (*n* = 10) rated the app as (very) unique compared to current websites available for individuals affected by HD, and indicated that using the app would be (very) helpful to them. One premanifest HDGEC commented: *“I think it looks nice and it would help me a lot if I did not know much about Huntington’s. I would also recommend my friends to check out this app so they can get a better idea [about HD].”*

With regard to the additional concepts that resulted from the co-creation sessions with other countries, all participants were (very) positive about the digital mock-ups of the online walk-in hour and the possibility to book an appointment with an HCP. The majority of participants were also (very) positive about the contact form (*n* = 13) and live chat (*n* = 12). A partner of a manifest HDGEC wrote about the additional value of the live chat: *“I definitely would have used this [live chat] during the time I had doubts about my partner’s behavior and found it difficult to find help.”* Participants indicated the additional features to be an “easily accessible” way to “quickly” and “directly” get in touch with an HCP. The digital mock-ups encouraged them to list some important requirements to consider when implementing such features (e.g., expected technical difficulties once the disease progresses, staff costs, privacy/security issues, and creating clarity about the aim of these features).

Although the main focus during prototype testing was on the desirability and usability of its functions, some participants wrote down some points for improvements regarding the app’s content, these have been described in the Supplementary material.

#### 3.5.3 Develop second prototype

Based on the input from the other countries and the positive feedback from Dutch participants on the digital mock-ups, three of the interactive features were implemented in the second prototype, i.e., the online walk-in hour, appointment booking, and contact form. To enable implementation in a feasible way, the walk-in hour and appointment booking functionalities redirected to third party apps (i.e., Microsoft Teams and Calendly, respectively). The live chat was not implemented due to privacy and security regulations. No additions to the initial content structure and topics were made due to the strong focus on implementing the interactive features during this version.

The second prototype was shielded from public access by implementing a pin code to avoid raising expectations among the HD community, which was expected to happen if the prototype would have been made public.

#### 3.5.4 Evaluate second prototype

The second version of the prototype was tested by 15 of the Dutch participants that participated in earlier phases (i.e., four premanifest HDGECs, three manifest HDGECs, five partners of premanifest HDGECs, and three partners of manifest HDGECs). Twelve of them also tested the first version of the prototype.

The initial reaction of all participants to the second version of the app was (very) positive. All participants found the app, as well as the pin code (very) easy to use. One premanifest HDGEC wrote: “*Furthermore, I am very positive about this portal and I am certain that this meets a need*.”

With regard to the newly implemented features, all participants rated the walk-in hour and appointment booking feature as (very) positive and pleasant. Participants valued that these features invite individuals to seek help/counseling and that they could schedule an appointment or visit the walk-in hour whenever it suits them: “*Especially the possibility to get in touch from home is really great, especially for people who live far away or have difficulty walking*” (a premanifest HDGEC). More particularly, participants liked the description of the procedures of the walk-in hour, and the button to contact someone when no walk-in hour is scheduled (for instance a social worker who can direct incoming questions to the right HCP). The majority rated the walk-in hour and appointment booking tool to be very unique (*n* = 10 and *n* = 11 respectively) compared to other HD services. Participants found these features (very) easy to use (*n* = 14) and indicated that they would like to use these features either regularly or maybe/sometimes. Regarding the contact form feature, participants were also (very) positive about its pleasantness (*n* = 14) and ease of use (*n* = 13), yet found it only a bit unique compared to other available HD services (*n* = 9).

Some participants wrote down suggestions for the app’s improvement, which have been described in the Supplementary material.

#### 3.5.5 Refine second prototype

Refinements resulted in a final prototype (see Figure 6), in which the majority of the Dutch content was drafted and subsequently uploaded. Content was based on existing resources and checked by HD experts. Moreover, some additions proposed by participants during both prototype testing rounds were incorporated [i.e., more regional information on other HD centers, links to YouTube videos, instruction videos, alternative contact possibilities for when the interactive features are unavailable, warning notification on (not) sharing privacy-sensitive data, and a description of the aim of the interactive features]. Due to budget and time constraints, some major changes could not be implemented such as adding an indication of the waiting time in the walk-in hour waiting room or booking an appointment in the respective language (rather than in English), as these would require integrating these interactive features of third-party apps in the app itself.

Several name suggestions for the prototype’s working title “Huntington Service Portal” were made by the international team. Each country decided on a top 3 of names for the app (i.e., Huntington Support, Huntington Info, and Huntington Portal). Discussions with the international team led to renaming the app to “Huntington Support App” as the term “portal” was not considered that common in all countries and the app consisted of more than just information (i.e., “info”) alone.

## 4 Discussion

In this article, we described the exploration and development process of an eHealth application that aims to support the quality of life (QoL) of HD gene expansion carriers (HDGECs) and their partners across Europe. This is the first study that used the principles of human-centered design (HCD) to develop an eHealth service that ensures suitability with the needs and wishes of HDGECs, partners, and health care providers (HCPs) by actively involving them as end-users throughout each stage of the development process (i.e., from ideation to prototype). Our study provides a comprehensive documentation of the application of HCD, and how we adopted this approach in the context of HD. We described the iterative process of co-designing, evaluating and adjusting an eHealth application together with end-users in the context of different health care systems in the Netherlands, Germany, Czech Republic, Italy, and Ireland, in the midst of the COVID-19 pandemic. The resulting Huntington Support App prototype provides support and guidance in the form of a central online place with clear and reliable information on the disease, HD-related news and events, as well as direct contact possibilities with HCPs via an online walk-in hour or by scheduling an appointment.

### 4.1 Human-centered design for HD

While eHealth holds promise for HD, there has been limited research on this topic (19–24), especially regarding eHealth services designed in active collaboration with HDGECs and their partners. Participatory development is, however, strongly recommended for developing solutions to fit the end-users’ needs (18, 26, 31, 32) and is found to be beneficial in other neurodegenerative and neurological diseases (e.g., dementia, Parkinson’s disease, and multiple sclerosis) (33–35). In line with this, our findings illustrate that such a participatory development process, and an HCD approach in particular, is valuable in developing and tailoring an eHealth solution for a disease as rare and complex as HD. As the implications of a neurodegenerative disease like HD vary widely among individuals, a holistic systemic approach toward human needs is paramount to ensure that solutions fit the dynamics of the healthcare system users are part of (36). Hence, we involved patients and partners in varying disease stages, HCPs, and an international family patient expert panel (FPEP) to capture and support the full scope of HD.

Moreover, both HDGECs and partners were highly motivated for study participation and talked openly about their experiences during the individual and group-based sessions. We were able to gain a deeper level of understanding of their experiences (45, 46) by using generative techniques like sensitizing assignments and journey mapping (37) in addition to the more conventional techniques of interviews and focus groups. We also included participants in the identification of technical solutions, co-creation of concepts and evaluations of the prototypes. These sessions contributed to valuable findings in terms of content and navigation ideas, most of which we were able to implement directly. Our HCD approach was also well received by the FPEP and the advisory board.

Given the advances in the field of design thinking, we believe that the term “user-centered design” as previously used in our study protocol (37), does not fully cover all the steps of our development process. In addition to end-users testing our eHealth solution, we took a broader, more inclusive approach to user-centered design by focusing on the “human” behind the product rather than the “user” of a product. By emphasizing and gaining a comprehensive understanding of the end-users’ personal experiences and their context (47), our study reflects a novel case of HCD research in HD (48).

### 4.2 Dealing with differences in needs across end-user groups and countries

Throughout the development of the Huntington Support App, we included a variety of perspectives from different end-user groups (premanifest/manifest HDGECs and partners, as well as HCPs). This was considered important given the variety in needs and symptoms experienced across disease stages. At the same time, integrating a variety of perspectives into a single eHealth solution is challenging. For instance, in contrast to the need for information addressed by HDGECs and their partners, the participating Dutch HCPs mentioned that a lot of services for HD are already available and adding something new might be a challenge. They, for example, mentioned online training videos, information resources, and tele-care technologies. Moreover, needs and wishes varied across the participating countries as well. While in the Netherlands end-users mainly focused on information and contact possibilities, HD-experts from the other countries expressed a higher need for interactive elements in the app.

Co-designing and developing an app is a time and resource-intensive process, especially amid a pandemic. During our development process, we learned that the needs and wishes of multiple end-user groups could not be met by a single eHealth solution. The development of multiple eHealth solutions should therefore be considered when defining several end-user groups. To make optimal use of our available resources (i.e., time and budget), we prioritized HDGECs and partners as the main end-user groups when moving along the development process due to their overlapping needs. The inclusion of a partner group is a key strength of this study as partners also experience impaired QoL (8–10) and often find it difficult to look after their own needs and experiences (10). Their perspectives should not be overlooked as partners often play an important role throughout the course of the disease. By actively involving the advisory board and co-creating functionalities together with HCPs from the participating countries, we ensured the inclusion of relevant clinical expertise and considered challenges that might arise throughout the course of the disease that could affect engagement of HDGECs later on (e.g., motor impairments, apathy).

Moreover, as multiple eHealth solutions could fit a single need, we had to prioritize which solutions to implement and which not. For instance, in case of facilitating contact, solutions could range from a list with contact details of care institutions/HCPs and peer groups to group chats and video-conferencing. Together with end-users, we decided which solution(s) to further develop and implement in the app considering contextual constraints. For example, we did not implement a live chat function as, next to security/privacy challenges, this would place a large responsibility on HCPs and also increase the expectation among users that they would receive an immediate response.

Despite the differences across end-user groups and countries, we were able to address a variety of the needs and wishes expressed, by designing a customizable set of functions and features that were considered beneficial in the care context of each country. This flexibility in design, together with the underlying content management system for each country, is of particular value as the app can be easily adapted and tailored to specific target groups and different healthcare systems/language regions, and extended with new features if new needs arise.

### 4.3 The Huntington Support App

With information and facilitating contact as key components of the app, the Huntington Support App prototype closely fits with the end-users’ wish for a central place with complete and correct information on HD, as well as opportunities for professional and informal support. With the addition of the interactive elements, the Huntington Support App also provides a low threshold for consulting an HCP by either visiting an online walk-in hour or by scheduling an appointment. This could in turn support early guidance and counseling in coping with HD. As suggested in a recent study on telehealth use in HD (24), we tailored the user interface to future HD-associated challenges. For example, with the future progression of the disease in mind, we specifically decided on a web-app format that is openly accessible to both HDGECs and partners as well as the larger HD community to facilitate accessibility of the app. In this way, individuals can access the app regardless of preferred device (e.g., mobile phone, tablet, and personal computer), its’ operating system or the location that someone is at. This circumvents potential difficulties with downloading or logging into the app once motor and/or cognitive decline sets in, and, at the same time, allows for the use of custom devices if they have a web browser.

The prototype of the Huntington Support App and its interactive elements were positively received by both Dutch HDGECs and partners. Only a slight majority rated the app as (very) unique compared to other services currently available for HD. This is not surprising, as our needs analysis and co-creation results indicated that although information and tools are indeed already available, these are not easily found and integrated for HD. Findability of information should therefore not be considered as a matter of course. This shows the importance of engaging end-users throughout the design process as, in our case, utilizing and integrating existing information and resources can accommodate the end-users’ needs and wishes rather than developing a new solution from scratch.

Regarding the interactive features of the app, privacy and security matters were a major concern. The combination of certain personal information (e.g., birth date and place of residence) can be easily identifiable in a disease as rare as HD. A secure app that guarantees the end-users’ privacy is therefore mandatory, yet securing such interactive features did not fit within the scope and budget for this project. Without tracking personal data, as is the case with our focus on information provision of the app, such issues are circumvented. To still be able to facilitate interactive features, we decided to redirect to third party apps that have their own privacy and security policies implemented.

### 4.4 Study limitations

Some limitations of this study should be addressed. First, we included a relatively well-functioning and motivated sample of HDGECs, thereby possibly omitting the needs and wishes of more severely affected HDGECs and/or people with fewer digital skills or who were less motivated for research participation. Efforts were made to include HDGECs with varying disease stages, gender, age, and digital literacy. By also including partners of HDGECs with different disease stages, as well as HCPs with relevant expertise and experience with HDGECs across these stages, we were able to ensure a wide range of different perspectives. We also incorporated different scenarios in the co-design preparations, which encouraged participants to think beyond their personal experiences. Other limitations mainly resulted from the contextual constraints (i.e., time, budget, and feasibility) posed by the COVID-19 pandemic. We believe that the resulting deviations from our study protocol (37) were minor and did not impact the study’s results due to the flexibility of our HCD approach. Involvement of the other countries amid a pandemic was especially challenging as each country and health care system was affected differently. Although the other participating countries were therefore involved to a lesser extent as initially planned (37), we believe that actively including HD experts from the participating sites as well as the FPEP was sufficient to adapt the app to each language and healthcare system. Due to the COVID-measures in place at the time, most study sessions were conducted online. Recent studies show that the data quality of online interviews and focus groups is comparable to that of in-person interviews (49–52). As most Dutch participants were able to connect easily to the videoconferencing platform and shared detailed personal experiences with us, we believe online sessions were both feasible and suitable for our population given the posed COVID-19 restrictions. At the same time, it allowed participants to get used to remote services, which fits well with their contribution to the development of such a service during this study as well as with the post-pandemic shift toward blended care (53) that likely increases the use of such services in the future.

### 4.5 Future directions

Although the present article focused on the development of the Huntington Support App prototype, future efforts should focus on an in-depth user experience and usability evaluation as well as pilot-testing the app in the different countries. Given the potential of the Huntington Support App, a feasibility study of the app is needed to identify implementation challenges that could be addressed in the future. For instance, an in-depth evaluation of the use and feasibility of certain features that are currently available via third-party apps (e.g., walk-in hour, appointment booking) is recommended prior to fully integrating such features within the app. These interactive features were recommended by HD experts from the project consortium and did not address the major needs and wishes of Dutch end-users in our HCD approach. Future research should investigate the value of such applications from both the expert and end-user perspective. Follow-up research should further focus on how to successfully implement the Huntington Support App, while at the same time taking privacy, security and other contextual issues (i.e., staff availability, updating the app’s content) into account. Important areas of focus include investigating barriers and facilitators to implementation [e.g., awareness and acceptance of the app, financial constraints (54)], as well as identifying key stakeholders for implementation, such as HD expertise centers but also general practitioners as they are typically the first point of contact when people access the Dutch healthcare system.

Moreover, as we were not able to focus on all expressed needs and wishes in the current prototype, future efforts could be directed toward addressing these. For instance, by further investigating the expressed needs of HCPs regarding strengthening the coordination and communication between HCPs both nationally and internationally. Given that HD is a rare and complex disease, international connection and collaboration between experts is of great value. Future developments should be directed at further amending the Huntington Support App prototype to the local HD care context and respective languages in each country. The needs and wishes of HCPs should be integrated to optimally align eHealth services to HCPs as end-users, as this will likely enhance the future adoption and implementation of eHealth services across Europe.

### 4.6 Conclusion

The present study demonstrates that a participatory HCD approach is valuable in developing and tailoring an eHealth application to the needs and wishes of HDGECs and their partners across Europe. This high level and active involvement of end-users is important to increase uptake and improve the usability and acceptability of eHealth services once implemented (18, 26–31, 55). As QoL maintenance is one of the main goals in HD care, the app could be a first step in supporting QoL by harnessing the power of eHealth, especially as effective interventions specifically targeting QoL are limited to date (7). The Huntington Support App has the potential to (partially) overcome challenges of time, distance, and costs in current HD care provision (32). Future efforts should focus on evaluating the app’s feasibility, as well as its effectiveness in supporting QoL while taking into account challenges that may arise during implementation (e.g., staff and reimbursement issues) (56). With these implications in mind, the Huntington Support App could contribute to increasing accessibility of HD care and enhancing QoL care across Europe. Low-threshold access to health care and reliable information can be considered pivotal in the management of long-term complex conditions (57). With our detailed description of the lessons learned while developing the app in the midst of the COVID-19 pandemic and how we adopted a HCD approach when designing eHealth for HD, we believe that our results are of great relevance for future co-design initiatives of telehealth services aimed at enhancing supportive care service for other rare neurodegenerative diseases worldwide.

## Data availability statement

The raw data supporting the conclusions of this article will be made available by the authors, without undue reservation, upon reasonable request.

## Ethics statement

The medical research ethics committee of Leiden Den Haag Delft cleared this study for ethics in the Netherlands (file number: N20.013). For the other participating countries, ethical approval was obtained by the local medical ethics committees at the respective sites. These were: Germany: Ethics committee of Ulm University; Czech Republic: Ethics committee of the General University Hospital Prague; Italy: Ethics committee of the Casa Sollievo della Sofferenze; Ireland: Faculty of Health Sciences Ethics Committee of Trinity College Dublin. The studies were conducted in accordance with the local legislation and institutional requirements. The participants provided their written informed consent to participate in this study.

## HEALTHE-RND consortium

The Netherlands: Niels H. Chavannes, Eline Meijer, Anne-Wil Heemskerk, Erik van Duijn, Susanne T. de Bot, Pearl J. C. van Lonkhuizen, Niko Vegt, Leanne Slutter, Stephanie Feleus, Esther C. Arendts, and Amy Putman; Germany: G. Bernhard Landwehrmeyer, Alzbeta Mühlbäck, Wiebke Frank, and Franziska Steck; Czech Republic: Jiří Klempíř, Romama Konvalinková, Eva Bezuchová, Kristýna Dolečková, Olga Klempířová, Jan Roth, and Olga Ulmanová; Italy: Ferdinando Squitieri, Sabrina Maffi, Giulia Giancaterino, Chiara Di Giorgio, Barbara D’Alessio, and Melissa Casella; Ireland: Jennifer Hoblyn, Muthukumaran Thangaramanujam, Tom Burke, and Emer O’Malley; United Kingdom: Stephen McKenna, Ian McKenna, Jeanette Thorpe, Ellie Johnstone, Isobel Spray, Mariusz Grzeda, Ramona Moldovan, Peter Foley, and Jacqueline Kerr.

## Author contributions

PL: Writing – review & editing, Writing – original draft, Validation, Formal analysis. A-WH: Writing – review & editing, Supervision, Formal analysis. EM: Writing – review & editing, Supervision, Funding acquisition, Formal analysis, Conceptualization. ED: Writing – review & editing, Supervision. SB: Writing – review & editing, Supervision. JK: Writing – review & editing, Funding acquisition, Conceptualization. GL: Writing – review & editing, Funding acquisition, Conceptualization. AM: Writing – review & editing, Funding acquisition, Conceptualization. JH: Writing – review & editing, Funding acquisition, Conceptualization. FS: Writing – review & editing, Funding acquisition, Conceptualization. NC: Writing – review & editing, Funding acquisition, Conceptualization. NV: Writing – review & editing, Supervision, Formal analysis.

## Footnotes

^1^ https://apphaus.sap.com/resource/scenes
