# Supplementary material for: Development of the Huntington Support App (HD-eHelp study): a human-centered and co-design approach
Source: Front Neurol. 2024 Jul 1;15:1399126. doi: 10.3389/fneur.2024.1399126 (PMC11246862; doi:10.3389/fneur.2024.1399126)
Supplement: Supplementary file 1 [file Table_1.DOCX]

**Supplementary Information**

This supplementary file contains extra information that complements the main text of the manuscript. The following sections provide additional details on: 1) the translation from common themes into design goals (3.4.1); 2) the evaluation of the first prototype and additional concepts (3.5.2); and 3) the evaluation of the second prototype (3.5.4).

***Translate common themes into design goals (3.4.1)***

In addition to the design goal of ‘early guidance’, many people only come into view when they start to experience complaints and as a result, they often already have lived with their complaints and/or family history for a longer time. However, there is sufficient knowledge and expertise available to guide people from the moment they come into contact with HD. In order to get people into a guided process at an early stage, it is important that the contact has a low threshold. Digital resources can play a major role in this. As people’s acceptance and understanding of the disease may vary strongly, eHealth solutions need to provide carefully layered and structured information, so that users are in control of what they want to know and what not.

With regard to the design goal of ‘navigating through the knowledge landscape’, patients and partners sometimes feel misunderstood because information about the disease is often too generic or extreme in relation to their own situation. Digital tools can be used to, for example, provide a clear navigation structure that allows users to take control of which information is relevant to them at what time. People unfamiliar with the disease should receive a nuanced story about HD rather than the extreme stories that often circulate. The basic principle in this design goal is tailored information. eHealth solutions should distinguish between theoretical and practical information and it must be clear for which target group the information is intended.

The goal of providing ‘counseling in coping with HD’ through digital tools came from the interviews, in which participants talked about all kinds of ways in which they deal with HD. Examples forwarded by participants included recording the care path and wishes concerning end-of-life, contributing to HD trials and campaigns, keeping track of information about treatment methods, discussing concerns and experiences with others, and enjoying the moment. Digital tools could support these coping strategies by offering low-threshold contact with HCPs, as people often feel alone with their questions yet at the same time feel that they do not have sufficient reason to make an appointment with an HCP.

Regarding the goal of ‘dealing with symptoms’, a central point would be desirable where the most commonly used tools for HD are gathered together, such as planning and notification applications for daily life activities or a source of information for informal carers. Referral to specific tools for certain complaints (e.g., adapted cutlery, walking aids, specific shoes) could also help with finding suitable tools to deal with particular symptoms. Acceptance of one's own illness and possibly the insight of the partner are important here.

***Evaluate first prototype and additional concepts (3.5.2)***

Although the focus during prototype testing was on desirability and usability of its functions, some participants also mentioned suggestions for the app’s content, including increasing the representativeness of other (regional) HD expertise centers and including people living with the risk of HD as a target audience. Moreover, some participants proposed to include information on relevant topics that were missing at the time, such as writing a will, euthanasia, and pre-implantation genetic diagnosis, as well as by linking to tv shows or YouTube videos about HD-related topics. During the online feedback sessions (attended by one premanifest and two manifest HDGECs), some more additions to the app were suggested, including a short instruction on how to use the app on the home page, instruction videos, and a description of what people can expect from the additional features and alternative contact possibilities when the features are (temporarily) unavailable.

***Evaluate second prototype (3.5.4)***

During the second prototype test, additional suggestions for the app’s improvement were made by some participants, including a description of how to return to the app once redirected to another site, and implement a clear warning notification in the interactive features on (not) sharing privacy-sensitive data. With regard to the interactive features, some points for improvement were mentioned (e.g., appointment scheduling in Dutch rather than English via a third-party app, including an indication of the waiting time and the possibility to chat rather than video calling during the walk-in hour). Moreover, some proposed to include a clear description about the aim of the interactive features: *“It must be very clear what the function of the walk-in hour is. Is it about informing people about various processes and steps that cannot be found on the website? Or is it an overlap with video calling function of [Huntington expertise center]?”* (a premanifest HDGEC). During the online feedback sessions (attended by two premanifest and two manifest HDGECs), additional feedback was discussed (e.g., adding more content and regional information, avoid overlap with other HD websites and opening new tabs in the browser, and consider an introductory tutorial on the use of the app).

**Supplementary Table 1.** Sociodemographic characteristics and recruitment rates of end-users from participating countries in phase 1

|  | Germany | | | Italy | | | Ireland | | | Czech Republic | | |
| --- | --- | --- | --- | --- | --- | --- | --- | --- | --- | --- | --- | --- |
|  | HDGECs  (N = 6) | Partners  (N = 5) | HCPs  (N = 0) | HDGECs  (N = 6) | Partners  (N = 6) | HCPs  (N = 0) | HDGECs  (N = 7) | Partners  (N = 7) | HCPs  (N = 6) | HDGECs  (N = 0) | Partners  (N = 0) | HCPs  (N = 0) |
| Age (mean; range) | 45; 26-59 | 63; 55-76 | - | 32; 20-42 | 42; 28-54 | - | 43; 35-51 | 52; 34-71 | 43; 26-69 | - | - | - |
| Gender (n (%)) |  |  |  |  |  |  |  |  |  |  |  |  |
| Male | 5 (83) | 1 (20) | - | 2 (33) | - | - | 1 (14) | 4 (57) | 4 (67) | - | - | - |
| Female | 1 (17) | 4 (80) | - | 4 (67) | 6 (100) | - | 6 (86) | 3 (43) | 2 (33) | - | - | - |
| HD stage affected individual (n (%)) |  |  |  |  |  |  |  |  |  |  |  |  |
| Premanifest | 3 (50) | 2 (40) | - | 3 (50) | 3 (50) | - | 5 (71) | 3 (43) | - | - | - | - |
| Manifest | 3 (50) | 3 (60) | - | 3 (50) | 3 (50) | - | 2 (29) | 4 (57) | - | - | - | - |
| Living situation (n (%)) |  |  |  |  |  |  |  |  |  |  |  |  |
| Together with partner | 2 (33) | 5 (100) | - | 3 (50) | 6 (100) | - | 5 (71) | 7 (100) | - | - | - | - |
| Alone | 1 (17) | - | - | - | - | - | - | - | - | - | - | - |
| Other (e.g., with child,  friend, parent) | 3 (50) | - | - | 3 (50) | - | - | 2 (29) | - | - | - | - | - |
| Time since genetic test in years (mean; range) | 11; 2-21 | - | - | 6; 1-12 | - | - | 9; 3-19 | - | - | - | - | - |
| Profession (n (%)) |  |  |  |  |  |  |  |  |  |  |  |  |
| Occupational therapist | - | - | - | - | - | - | - | - | 1 (17) | - | - | - |
| Nurse | - | - | - | - | - | - | - | - | 3 (50) | - | - | - |
| Psychiatrist | - | - | - | - | - | - | - | - | 1 (17) | - | - | - |
| Psychologist | - | - | - | - | - | - | - | - | 1 (17) | - | - | - |

*Note*. HDGECs: Huntington’s disease gene expansion carriers; HCPs: health care providers; N: number of participants; HD: Huntington’s disease. Numbers and percentages are rounded to the nearest whole number. Due to feasibility and time constraints posed by the COVID-19 pandemic, these numbers differ from the original estimated sample size (37).
